# Monitoring Circulating γδ T Cells in Cancer Patients to Optimize γδ T Cell-Based Immunotherapy

**DOI:** 10.3389/fimmu.2014.00643

**Published:** 2014-12-17

**Authors:** Hans-Heinrich Oberg, Christian Kellner, Matthias Peipp, Susanne Sebens, Sabine Adam-Klages, Martin Gramatzki, Dieter Kabelitz, Daniela Wesch

**Affiliations:** ^1^Institute of Immunology, Christian-Albrechts-University of Kiel, Kiel, Germany; ^2^2nd Medical Department, Division of Stem Cell Transplantation and Immunotherapy, Christian-Albrechts-University of Kiel, Kiel, Germany; ^3^Institute for Experimental Medicine, Christian-Albrechts-University of Kiel, Kiel, Germany

**Keywords:** monitoring, human, γδ T cells, pancreatic ductal adenocarcinoma, bispecific antibodies, phosphorylated antigens, aminobisphosphonate

## Abstract

The success of γδ T cell-based immunotherapy, where the cytotoxic activity of circulating γδ T lymphocytes is activated by nitrogen-containing bisphosphonates (n-BP), or possibly by bispecific antibodies or the combination of both, requires a profound knowledge of patients’ γδ T cells. A possible influence of radio- or chemotherapy on γδ T cells as well as their reported exhaustion after repetitive treatment with n-BP or their lack of response to various cancers can be easily determined by the monitoring assays described in this perspective article. Monitoring the absolute cell numbers of circulating γδ T cell subpopulations in small volumes of whole blood from cancer patients and determining γδ T cell cytotoxicity using the Real-Time Cell Analyzer can give a more comprehensive assessment of a personalized tumor treatment. Possible future directions such as the combined usage of n-BP or phosphorylated antigens together with bispecific antibodies that selectively target γδ T cells to tumor-associated antigens, will be discussed. Such strategies induce expansion and enhance γδ T cell cytotoxicity and might possibly avoid their exhaustion and overcome the immunosuppressive tumor microenvironment.

## Introduction

Human γδ T cells (γδTc) represent a small subset (1–10%) of CD3^+^ T lymphocytes with several unconventional features. Similar to antigen presenting cells (APC), γδTc can phagocytose and present soluble antigens to CD3^+^ αβ T cells (1, 2). Additionally, γδTc can induce the maturation of dendritic cells (DCs), and kill various tumor cells in a HLA-independent manner (3, 4). Thus, there is a substantial interest in γδTc in the context of T cell-based immunotherapeutic strategies (5, 6). Several pilot studies have described a partial success of γδ T cell-based immunotherapy in different types of cancer after the application of aminobisphosphonates (n-BP) or phosphorylated antigens (PAg) plus IL-2 *in vivo* or after repetitive transfer of *in vitro* expanded Vδ2-expressing γδTc (7–10). Although γδ T cell-based immunotherapy has delivered promising results, sustained stimulation of Vδ2 γδTc by n-BP or PAg often leads to Vδ2 T cell exhaustion (8, 11, 12). Additionally, a low number of functionally unresponsive γδTc has been described in patients with chronic lymphocytic leukemia or multiple myeloma (13–15). Novel bispecific antibodies (with concomitant specificity for epitopes on both γδTc and tumor cells) provide a tool to enhance cytotoxic activity of γδTc against cancer cells by selectively targeting γδTc to antigens expressed by tumor cells (16). Additionally, independent of previous immunotherapeutic strategies and prior to the application of a γδ T cell-based immunotherapy, it is mandatory to analyze the number and functional capacity of patients’ γδTc in a simple manner. This article demonstrates that the analysis of absolute cell numbers of circulating γδTc from patients as well as the determination of the cytotoxic capacity against tumor cells of interest can give a better assessment of subsequent personalized tumor treatment.

## Monitoring of Absolute Cell Numbers

**Figure 1:**
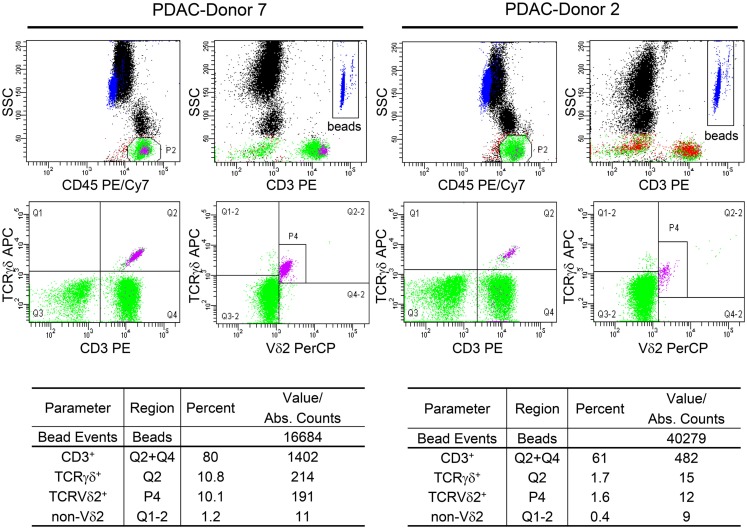
**Determination of the absolute cell number of circulating γδ T cells and their subsets in blood of PDAC patients**. Fifty microliters whole blood samples from PDAC patients were stained with the indicated mAb in BD Trucount™ Tubes. These mAbs were previously titrated and a final concentration of 2–5 μg/ml was used. The mAb cocktail can be prepared in advance in bulk. The BD Trucount™ tubes contain lyophilized pellets that dissolve after adding liquid, thereby releasing a known number of fluorescent beads. Two hundred microliters of BD Lysing buffer was added to lyse red blood cells. To distinguish lymphocytes and beads from granulocytes and monocytes, an appropriate gate was set on CD45^+^ cells or beads using side scatter and CD45 or CD3 expression, respectively (upper panel). The ratio of the event number in the bead gate was compared to the total number of beads originally in the tube. The absolute cell number (Abs. Counts) of CD3^+^ (CD3), CD3^+^ TCRγδ^+^ (γδ), TCRγδ^+^ TCRnon-Vδ2^+^ (non-Vδ2), and TCRγδ^+^ TCRVδ2^+^ (Vδ2) within CD45^+^ lymphocytes was calculated as follows: (cells/microliter of whole blood) = [(events of cells of interest)/(ratio of acquired bead events to total beads in pellet)]/50 μl. Two representative determinations (PDAC-Donor 7 and 2) of 21 are shown, as are the percentages of the different cell populations.

The monitoring system that uses the BD Multitest 6-color TBNK (M6T) Reagent with BD Trucount™ Beads (http://www.bd.com/resource.aspx?IDX=17743, BD Biosciences, San Jose, CA, US) allows determination of absolute cell numbers of αβ T and B lymphocytes and NK cells as well as CD4^+^ and CD8^+^ T cell subsets (17, 18). Since γδ T lymphocytes and their subpopulations are not detected by the M6T, we adapted γδTc staining from the BD Trucount™ Tube technical data sheet (version 8/2010) as follows: 50 μl whole blood from cancer patients were stained with anti-CD45-PE/Cy7 (clone HI30), CD3-PE (clone SK7) pan-TCRγδ-APC (clone 11F2, customized) (all from BD Biosciences, Heidelberg, Germany), and Vδ2-PerCP (clone B6, Biolegend, Fell, Germany) mAbs and occasionally with Vδ1-FITC mAb (clone TS8.2, Thermo Fisher Scientific, Germany) in BD Trucount™ Tubes as described (16). After staining, red blood cells were lysed with 200 μl BD Lysing buffer and analyzed using the FACS Canto flow cytometer and FACS Diva software (both from BD Biosciences). For two representative donors, the absolute numbers of total γδTc as well as Vδ2 and non-Vδ2 subsets are shown (Figure 1). Moreover, cells can be stained with anti-Vδ1 mAb labeled with an additional fluorochrome (data not shown).

Certainly, other bead-based detection systems could be used alternatively to determine absolute cell numbers. Importantly, however, these strategies must allow this determination from a small volume of patient’s blood.

In addition, a possible influence of radio- or chemotherapy on circulating immune cell numbers can be easily determined by this monitoring system. For instance, our own data reveal that the absolute number of Vδ2 γδTc in a cohort of 10 breast cancer patients receiving chemotherapy did not differ from age-matched breast cancer patients without treatment (Adam-Klages et al., unpublished data). Moreover, in a cohort of 41 patients with pancreatic ductal adenocarcinoma (PDAC, stage pT3–4, pN0–1, L0–1 and V0–1), we recently observed that the decrease in absolute numbers of Vδ2 γδTc did not correlate with cancer stage/progression, but rather with patient age (16).

While determination of the absolute γδ T cell numbers and that of their subsets provides no information about their cytotoxic capacity, this can be addressed in an additional functional assay.

## Determination of Cytotoxic Capacity

We recently examined the functional capacity of γδTc from patients with PDAC (16). PDAC is a highly aggressive gastrointestinal malignancy characterized by the presence of desmoplastic stromal microenvironment where conventional treatment approaches including surgery, chemotherapy, and/or radiation are often not effective (19). The observed decrease in absolute Vδ2 T cell numbers in untreated patients with advanced PDAC is attributable to age, not disease status, as similar numbers were found in age-matched healthy controls (16). In an attempt to avoid Vδ2 T cell exhaustion through repetitive n-BP stimulation and overcome the immunosuppressive activity of PDAC stromal cells on cytotoxic γδ T cells, novel bispecific antibodies such as [Her2xCD3] and [(Her2)_2_xVγ9] were designed. [(Her2)_2_xVγ9] is specific for Vγ9 on γδTc (associated with Vδ2) and for human epidermal growth factor receptor HER2/neu overexpressed on PDAC, breast, and prostate cancer cells. The [(Her2)_2_xVγ9] tribody design allows monovalent binding to γδTc and bivalent HER2-targeting, which enhances avidity to the tumor cell and thereby increases cytolytic activity. Both bispecific antibodies selectively target γδTc to tumor antigens, thereby enhancing the cytotoxic activity of γδTc *in vitro* as well as *in vivo* in a PDAC grafted SCID-Beige mouse model (16).

In previous studies, we usually examined the functional capacity of γδ T cell lines or freshly isolated γδ Tc. Aiming to simplify handling of cells from patients with a low γδ T cell number in the following experiments, we investigated the functional capacity of cytotoxic γδTc within PBMC. We observed that the functional cytotoxic activity of circulating γδTc from patients can be determined in as few as of 1–2 × 10^6^ PBMC, readily obtainable from 2 to 4 ml of patients’ blood. We analyzed blood from 21 patients with PDAC after obtaining their informed consent and relevant institutional review board approvals (code number: D401/14). As a read out system for cytotoxic activity of γδTc within freshly isolated PBMC, the real-time cell analyzer (RTCA) single-plate system (ACEA, San Diego, CA, USA) was used. RTCA measures the impedance of adherent tumor cell monolayers, but not of suspended cells such PBMCs with electronic sensors. The measurement of impedance in arbitrary cell index units reflects changes in cellular parameters of tumor cells, which allows monitoring of cellular events in real time without the incorporation of labels over time periods of several days. The loss of impedance correlates with the γδ T cell-mediated lysis of tumor cells (16). A further advantage of measuring impedance over an extended time is that it enables us to observe whether tumor cells can regenerate when lysis is incomplete.

**Figure 2:**
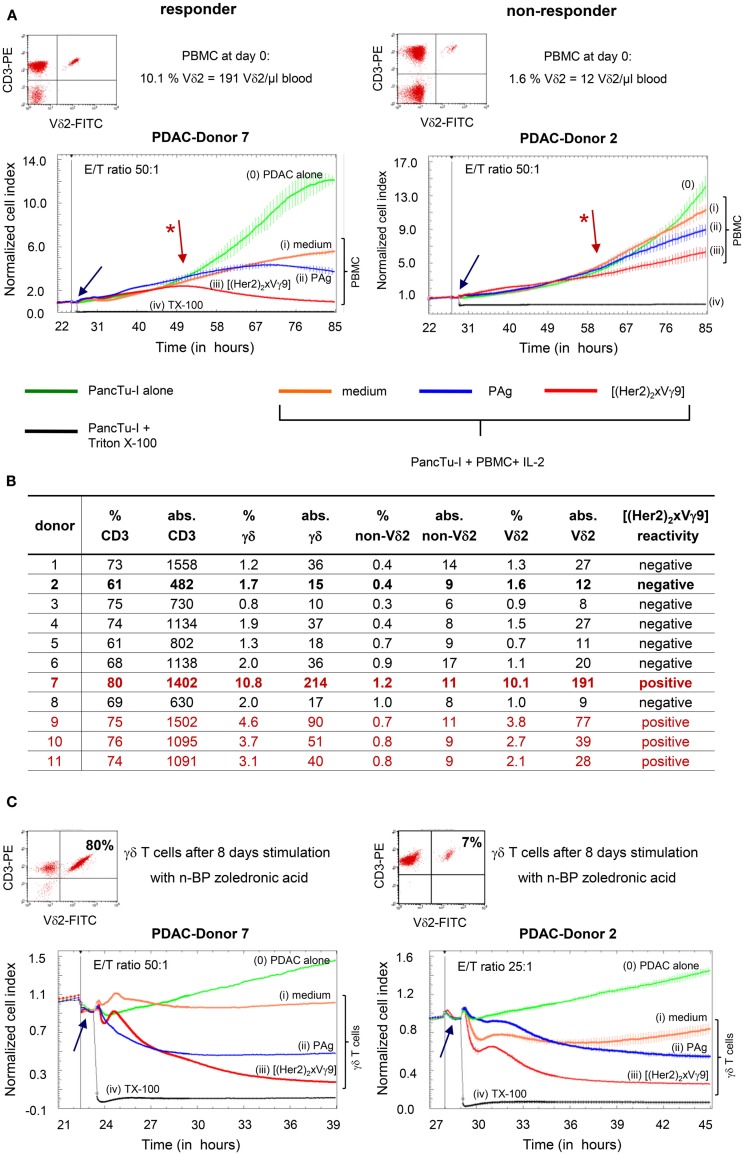
**Correlation between absolute cell number and functional capacity of Vδ2 T cells**. **(A)** Flow cytometric analysis of CD3^+^ Vδ2^+^ γδTc within PBMC, and RTCA of PBMC from two representative donors (Donors 7 and 2) of 21; **(B)** list of the relative and absolute numbers (abs.) of CD3, γδ, Vδ2, and non-Vδ2 T cells in whole blood from 11 representative PDAC patients out of 21 as well as reactivity to the tribody; **(C)** flow cytometric analysis of selective expansion of CD3^+^ Vδ2^+^ γδTc after PAg-activation within PBMC for 8 days, and RTCA with these short-term expanded γδTc from Donors 7 and 2. Two representative donors of 21 are shown. **(A,C)** For RTCA, 5 × 10^3^ PDAC cells (PancTu-I) were cultured in 10% FCS RPMI medium for 24–27 h on an E-plate covered at the bottom with electronic sensors that measure the impedance of the cells expressed as an arbitrary unit called cell index (CI). The CI was analyzed every 5 min to determine adherence and thus cell growth. Since the initial adherence in different wells can differ slightly, the CI was normalized to 1 shortly before the time of addition of suspended cells ± substances (vertical black line). After 24–27 h, PDAC cells were treated again with medium [green line (0)] or with PBMC **(A)** or short-term expanded γδTc **(C)** together with medium [orange line (i)], 300 nM PAg BrHPP [dark blue line (ii)], or 1 μg/ml [(Her2)_2_xVγ9)] [red line (iii)] at the indicated E:T ratio over the indicated time. As a positive control for maximal lysis, PDAC cells were treated with Triton X-100 [TX-100, black line (iv)]. The addition of substances, PBMC or expanded γδTc is indicated by the blue arrow. CI was then measured every minute for analysis of precise cytotoxicity time point for >15 to 55 h as indicated. The loss of tumor cell impedance and thus a decrease of the Normalized CI correlates with γδ T cell-mediated lysis of PDAC cells. The red arrow with the * points out the initiation of cytotoxicity. The average of triplicates and standard deviation were calculated; one representative experiment is shown.

To ensure adherence of tumor cells, PDAC cells were cultured for 24–27 h in RTCA plates before the addition of γδTc alone with or without additional substances. Thereafter, PDAC cells were still cultured alone or together with PDAC patient-derived PBMC in (i) medium, (ii) PAg such as bromohydrin-pyrophosphate (BrHPP), or (iii) [(Her2)_2_xVγ9]. During the extended time course, we observed that γδTc within PBMC required almost 24–36 h after initial stimulation to exert their cytotoxic capacity (Figure 2A, red arrow with a star). Moreover, we observed that [(Her2)_2_xVγ9] triggered tumor cell lysis more efficiently than PAg in 30% of PDAC patient samples (Figure 2A, responder), while neither substance was effective in 70% of patient samples (Figure 2A, non-responder). The unexpected cytotoxicity against PDAC cells in the absence of a stimulus (medium, orange line) is likely due to the reactivity of NK cells in the presence of IL-2 (Figure 2A), because additional experiments with untouched, freshly isolated γδTc demonstrated that cytotoxic activity of γδTc is not induced by IL-2 alone (16).

Regarding the absolute Vδ2 T cell numbers presented in Figure 2B (table), we correlated the unresponsiveness of the majority of the tested patient samples [negative [(Her2)_2_xVγ9] reactivity] with their low initial Vδ2 T cell number. PBMC from patients with more than 30 Vδ2^+^ γδTc/μl blood were responsive (responder in Figure 2A and “positive” in Figure 2B), whereas in samples with <30 Vδ2^+^ γδTc/μl blood, no induction of cytotoxic activity to PAg or [(Her2)_2_xVγ9] stimulation was observed (non-responder in Figure 2A and “negative” in Figure 2B).

The weak capacity of bispecific antibodies to induce γδ T cell proliferation could explain the observed unresponsiveness to [(Her2)_2_xVγ9]. Therefore, PBMC from the same patients were stimulated with the PAg BrHPP or, as presented in Figure 2C, with n-BP zoledronic acid for 7–14 days. Although the responder cells expanded to 80% γδTc in culture, while non-responders comprised only 7% after n-BP stimulation, this small population of non-responders exhibited nearly the same degree of cytotoxicity as responders after re-stimulation with [(Her2)_2_xVγ9], despite the lower effector/target ratio (Figure 2C).

Taken together, our results demonstrate that prior analysis of absolute circulating cell numbers of immune cell subsets as well as determination of their cytotoxic capacity against tumor cells of interest may provide a better assessment of whether a particular personalized tumor treatment will be effective.

## What Can We Learn from This Monitoring System?

γδ T cell monitoring can provide an estimate for a potential treatment of cancer patients. Although knowledge of the functional capacity of γδTc within PBMC does not provide information about their migration and infiltration into the tumor, characterization of these circulating γδTc is useful since they are activated by intravenous n-BP or PAg administration (8, 10). In clinical trials where γδTc were repetitively activated with n-BP or PAg together with low-dose IL-2, effects on tumor growth were observed; however, this was associated with exhaustion, anergy, or depletion of γδTc due to repetitive stimulation (8, 11, 12). In light of these observations, it is necessary to optimize cytotoxic activity, which can be achieved with bispecific antibodies such as the tribody [(Her2)_2_xVγ9]. Adoptive transfer of γδTc with [(Her2)_2_xVγ9] and IL-2 significantly reduced growth of pancreatic tumors grafted into SCID-Beige mice in comparison to adoptively transferred γδTc together with n-BP and IL-2 (16).

Vδ2 γδTc used for adoptive transfer are cells within PBMC that are initially activated with n-BP or PAg plus IL-2 (7, 20). Such initial activation with n-BP or PAg plus IL-2 causes selective Vδ2 T cell-expansion, while [(Her2)_2_xVγ9] does not induce strong proliferation of γδTc (unpublished data). Independently of the proliferative response of γδ Tc, the cytotoxic activity of PAg or n-BP expanded Vδ2 T cell lines can be significantly enhanced after re-stimulation with [(Her2)_2_xVγ9]. Moreover, the addition of [(Her2)_2_xVγ9] did not induce cell death of Vδ2 T cells, in contrast to restimulation of Vδ2 T cell lines with PAg (unpublished data). Thus, [(Her2)_2_xVγ9] provides a tool to further enhance cytotoxic activity of adoptively transferred γδTc, whereas PAg or n-BP failed because they induce cell death in almost half of the activated cells (unpublished data).

The observation that the majority of elderly people has a low frequency of γδTc hampers the expansion of autologous γδTc required for adoptive transfer. Considering these challenges, one might suggest adoptively transferring allogeneic or haploidentical γδTc from (younger) healthy donors or activating γδTc within PBMC *in vivo* with bispecific antibodies (21–23). To investigate the effect of bispecific antibodies on unstimulated γδTc, we monitored whether [(Her2)_2_xVγ9] can induce cytotoxic activity in γδTc within PBMC. As described above, no or weak responses to [(Her2)_2_xVγ9] were obtained with PBMC from PDAC donors with a lower frequency of Vδ2 γδTc (non-responder), whereas PBMC with a higher Vδ2 γδ T cell frequency responded to [(Her2)_2_xVγ9] resulting in enhanced cytotoxicity (responder) (Figure 2A). Interestingly, n-BP- or PAg-mediated enrichment of non-responder γδTc within PBMC for 7–14 days led to enhanced cytotoxic activity after restimulating the cells with [(Her2)_2_xVγ9] (Figure 2C).

The validity of this monitoring system to determine γδ T cell-reactivity within PBMC needs to be confirmed in patients undergoing γδ T cell-targeting therapy. Based on our experience, one might suggest initially administration of n-BP together with IL-2 in cancer patients to induce proliferation of Vδ2 γδTc followed by treatment with bispecific antibodies engaging γδTc plus IL-2 in order to avoid the Vδ2 T cell exhaustion observed in patients mediated by repetitive application of n-BP plus IL-2.

## What are the Benefits of Combining γδ T Cell-Based Immunotherapy with Bispecific Antibodies?

Therapeutic antibodies such as rituximab (anti-CD20 mAb) and trastuzumab or pertuzumab (both anti-HER2 mAb) as well as different combined therapies have clearly improved the treatment outcome of patients with B-cell lymphoma or breast cancer, respectively (24, 25). Furthermore, combining these therapeutic antibodies with γδ T cell-based immunotherapy seems very promising. Rituximab enhanced cytotoxic activity of *ex vivo* expanded CD16^+^ (FcRγIII) γδTc against CD20^+^ chronic lymphocytic leukemia, while Trastuzumab increased γδ T cell cytotoxicity against HER2^+^ breast cancer cells (26).

The success of such therapeutic antibodies has inspired antibody engineers to improve the antibody efficacy. One promising approach to enhance cytotoxicity and selectively target T cells to tumor-associated antigens is based on the usage of single-chain bispecific antibody constructs. One such construct is Blinatumomab with specificity for CD19 on lymphoma or leukemia and CD3 on T cells, which has proved efficient for the treatment of patients with hematological malignancies (27). The short half-life of only a few hours in serum requires continuous intravenous infusion of Blinatumomab, which induces an almost complete molecular response and prolonged leukemia-free survival in patients with minimal residual B-lineage acute lymphoblastic leukemia (28). The favorable characteristics of bispecific antibodies such as high specificity, high cytotoxic potential, and low immunogenicity, led us to design a bispecific antibody targeted to Vγ9 instead of CD3 and to HER2 expressed on several PDAC as well as on breast and prostate cancer, which could be easily replaced by another tumor target antigen of interest.

Of course, the question arises as to what differentiates bispecific antibodies with specificity for γδTc and those with specificity for CD3 T cells. For instance, a target group could be patients with advanced hematological malignancies (e.g., AML) who require allogeneic stem cell transplantation. A major advantage of γδ T cell-based immunotherapy is the HLA-independent killing of tumor cells, thereby reducing the risk of graft-versus-host disease often caused by alloreactive CD3^+^ αβ T cells (21, 22, 29, 30). A successful anti-tumor activity was described for patients with refractory hematological malignancies after adoptive transfer of haploidentical γδTc (23). Labeling *ex vivo* expanded haploidentical γδTc with bispecific antibodies could perhaps further enhance the cytotoxic capacity of these cells. A further advantage could be envisioned with respect to the innate lymphocyte capacity of γδTc to phagocytose and present antigens to αβ T cells, an activity that may be enhanced in the presence of a bispecific antibody. In the treatment of solid tumors, the initial administration of n-BP/IL-2 followed by infusion of bispecific antibody together with IL-2 could probably enhance cytotoxic activity of γδTc, which infiltrate several different tumor types at low frequency.

## Concluding Remarks

Bispecific antibodies have been designed in different formats. Clinical trials with bispecific antibodies such as Catumaxomab (TriomAb [EpCAMxCD3]), Ertumaxomab (Triomab [HER2xCD3]), and Blinatumomab (Bispecific T Cell Engager (BiTE) [CD19xCD3]) have delivered impressive therapeutic results. Additional clinical studies are certainly required to deeper evaluate and improve their therapeutic potential. Bispecific antibodies with specificity for CD3 enhance the cytotoxic potential of αβ as well γδ T cells. However, under certain circumstances, it would be desirable to activate only γδTc rather than a polyclonal population of T cells. For instance, CD8^+^ γδTc were presented at low frequency but at higher number than CD8^+^ αβ T cells in ductal epithelium and nearby stroma in PDAC tissues. This γδTc accumulation suggests an important role of γδTc in the immune response against PDAC, which is apparently suppressed by the pronounced immunosuppressive PDAC-microenvironment.

Together with the monitoring system described in this article, the tribody [(Her2)_2_xVγ9], which selectively targets γδTc and enhances their cytotoxic activity, provides a tool to determine the functional capacity of γδTc within the blood or within tumor-infiltrating T lymphocytes isolated from fresh tumor tissue of tumor patients. Whether bispecific antibodies targeting γδTc have the capacity to overcome the immunosuppressive stroma in PDAC patients, has yet to be investigated in further *in vivo* studies.

## Conflict of Interest Statement

The authors declare that the research was conducted in the absence of any commercial or financial relationships that could be construed as a potential conflict of interest.

## Abbreviations

BrHPP, bromohydrin-pyrophosphate; γδTc, γδ T cells; mAb, monoclonal antibody; n-BP, nitrogen-containing bisphosphonate; PAg, phosphorylated antigens; PDAC, pancreatic ductal adenocarcinoma; RTCA, real-time cell analyzer; TCR, T cell-antigen receptor.

## References

[1] Himoudi N Morgenstern DA Yan M Vernay B Saraiva L Wu Y Human γδ T lymphocytes are licensed for professional antigen presentation by interaction with opsonized target cells. J Immunol (2012) 188:1708–16. 10.4049/jimmunol.1102654 22250090

[2] Meuter S Eberl M Moser B. Prolonged antigen survival and cytosolic export in cross-presenting human γδ T cells. Proc Natl Acad Sci U S A (2010) 107:8730–5. 10.1073/pnas.1002769107 20413723 PMC2889313

[3] Devilder MC Allain S Dousset C Bonneville M Scotet E. Early triggering of exclusive IFN-γ responses of human Vγ9Vδ2 T cells by TLR-activated myeloid and plasmacytoid dendritic cells. J Immunol (2009) 183:3625–33. 10.4049/jimmunol.0901571 19710464

[4] Kabelitz D Kalyan S Oberg HH Wesch D. Human Vδ2 versus non-Vδ2 γδ T cells in antitumor immunity. Oncoimmunology (2013) 2:e23304. 10.4161/onci.23304 23802074 PMC3661159

[5] Fournié JJ Sicard H Poupot M Bezombes C Blanc A Romagne F What lessons can be learned from γδ T cell-based cancer immunotherapy trials? Cell Mol Immunol (2012) 10:35–41. 10.1038/cmi.2012.39 23241899 PMC4003170

[6] Wu YL Ding YP Tanaka Y Shen LW Wei CH Minato N γδ T cells and their potential for immunotherapy. Int J Biol Sci (2014) 10:119–35 10.7150/ijbs.7823 24520210 PMC3920167

[7] Bouet-Toussaint F Cabillic F Toutirais O Le GM Thomas DLP Daniel P Vγ9Vδ2 T cell-mediated recognition of human solid tumors. Potential for immunotherapy of hepatocellular and colorectal carcinomas. Cancer Immunol Immunother (2008) 57:531–9. 10.1007/s00262-007-0391-3 17764010 PMC11030195

[8] Dieli F Vermijlen D Fulfaro F Caccamo N Meraviglia S Cicero G Targeting human γδ T cells with zoledronate and interleukin-2 for immunotherapy of hormone-refractory prostate cancer. Cancer Res (2007) 67:7450–7. 10.1158/0008-5472.CAN-07-0199 17671215 PMC3915341

[9] Kobayashi H Tanaka Y Yagi J Osaka Y Nakazawa H Uchiyama T Safety profile and anti-tumor effects of adoptive immunotherapy using γδ T cells against advanced renal cell carcinoma: a pilot study. Cancer Immunol Immunother (2007) 56:469–76. 10.1007/s00262-006-0199-6 16850345 PMC11030814

[10] Meraviglia S Eberl M Vermijlen D Todaro M Buccheri S Cicero G In vivo manipulation of Vγ9Vδ2 T cells with zoledronate and low-dose interleukin-2 for immunotherapy of advanced breast cancer patients. Clin Exp Immunol (2010) 161:290–7. 10.1111/j.1365-2249.2010.04167.x 20491785 PMC2909411

[11] Braza MS Klein B. Anti-tumour immunotherapy with Vγ9Vδ2 T lymphocytes: from the bench to the bedside. Br J Haematol (2012) 160:123–32. 10.1111/bjh.12090 23061882

[12] Sicard H Ingoure S Luciani B Serraz C Fournié JJ Bonneville M In vivo immunomanipulation of Vγ9 Vδ2 T cells with a synthetic phosphoantigen in a preclinical nonhuman primate model. J Immunol (2005) 175:5471–80. 10.4049/jimmunol.175.8.5471 16210655

[13] Coscia M Vitale C Peola S Foglietta M Rigoni M Griggio V Dysfunctional Vγ9Vδ2 T cells are negative prognosticators and markers of dysregulated mevalonate pathway activity in chronic lymphocytic leukemia cells. Blood (2012) 120:3271–9. 10.1182/blood-2012-03-417519 22932792

[14] Wilhelm M Kunzmann V Eckstein S Reimer P Weissinger F Ruediger T γδ T cells for immune therapy of patients with lymphoid malignancies. Blood (2003) 102:200–6. 10.1182/blood-2002-12-3665 12623838

[15] Mariani S Muraro M Pantaleoni F Fiore F Nuschak B Peola S Effector γδ T cells and tumor cells as immune targets of zoledronic acid in multiple myeloma. Leukemia (2005) 19:664–70. 10.1038/sj.leu.2403693 15744346

[16] Oberg HH Peipp M Kellner C Sebens S Krause S Petrick D Novel bispecific antibodies increase γδ T-cell cytotoxicity against pancreatic cancer cells. Cancer Res (2014) 74:1349–60. 10.1158/0008-5472.CAN-13-0675 24448235

[17] Nicholson JK Stein D Mui T Mack R Hubbard M Denny T. Evaluation of a method for counting absolute numbers of cells with a flow cytometer. Clin Diagn Lab Immunol (1997) 4:309–13. 9144369 10.1128/cdli.4.3.309-313.1997 PMC170524

[18] Hensley-McBain T Heit A De Rosa SC McElrath MJ Andersen-Nissen E. Enumeration of major peripheral blood leukocyte populations for multicenter clinical trials using a whole blood phenotyping assay. J Vis Exp (2012) 411:23–36. 10.3791/4302 23007739 PMC3490252

[19] Sole CV Calvo FA Atahualpa F Berlin A Herranz R Gonzalez-Bayon L Role of radiotherapy in the chemotherapy-containing multidisciplinary management of patients with resected pancreatic adenocarcinoma. Strahlenther Onkol (2014). 10.1007/s00066-014-0759-1 25293727

[20] Bennouna J Bompas E Neidhardt EM Rolland F Philip I Galea C Phase-I study of Innacell γδ, an autologous cell-therapy product highly enriched in γ9δ2 T lymphocytes, in combination with IL-2, in patients with metastatic renal cell carcinoma. Cancer Immunol Immunother (2008) 57:1599–609. 10.1007/s00262-008-0491-8 18301889 PMC11030608

[21] Lamb LS Jr Lopez RD. γδ T cells: a new frontier for immunotherapy? Biol Blood Marrow Transplant (2005) 11:161–8. 10.1016/j.bbmt.2004.11.015 15744234

[22] Godder KT Henslee-Downey PJ Mehta J Park BS Chiang KY Abhyankar S Long term disease-free survival in acute leukemia patients recovering with increased γδ T cells after partially mismatched related donor bone marrow transplantation. Bone Marrow Transplant (2007) 39:751–7. 10.1038/sj.bmt.1705650 17450185

[23] Wilhelm M Smetak M Schaefer-Eckart K Kimmel B Birkmann J Einsele H Successful adoptive transfer and in vivo expansion of haploidentical γδ T cells. J Transl Med (2014) 12:45–50. 10.1186/1479-5876-12-45 24528541 PMC3926263

[24] Singh JC Jhaveri K Esteva FJ. HER2-positive advanced breast cancer: optimizing patient outcomes and opportunities for drug development. Br J Cancer (2014) 111:1888–98. 10.1038/bjc.2014.388 25025958 PMC4229628

[25] Wilson WH. Treatment strategies for aggressive lymphomas: what works? Hematology Am Soc Hematol Educ Program (2013) 2013:584–90. 10.1182/asheducation-2013.1.584 24319235

[26] Tokuyama H Hagi T Mattarollo SR Morley J Wang Q So HF Vγ9 Vδ2 T cell cytotoxicity against tumor cells is enhanced by monoclonal antibody drugs – rituximab and trastuzumab. Int J Cancer (2008) 122:2526–34. 10.1002/ijc.23365 18307255

[27] Nagorsen D Kufer P Baeuerle PA Bargou R. Blinatumomab: a historical perspective. Pharmacol Ther (2012) 136:334–42. 10.1016/j.pharmthera.2012.07.013 22940266

[28] Klinger M Brandl C Zugmaier G Hijazi Y Bargou RC Topp MS Immunopharmacologic response of patients with B-lineage acute lymphoblastic leukemia to continuous infusion of T cell-engaging CD19/CD3-bispecific BiTE antibody blinatumomab. Blood (2012) 119:6226–33. 10.1182/blood-2012-01-400515 22592608

[29] Bertaina A Merli P Rutella S Pagliara D Bernardo ME Masetti R HLA-haploidentical stem cell transplantation after removal of αβ+ T and B cells in children with nonmalignant disorders. Blood (2014) 124:822–6. 10.1182/blood-2014-03-563817 24869942

[30] Drobyski WR Majewski D Hanson G. Graft-facilitating doses of ex vivo activated γδ T cells do not cause lethal murine graft-vs.-host disease. Biol Blood Marrow Transplant (1999) 5:222–30. 10.1053/bbmt.1999.v5.pm10465102 10465102

